# 

**Published:** 1886-10-02

**Authors:** 


PRICE ONE PENNY.

No. 1, Vol. I.

October 2nd, 1886.

Per Annum, 6s. 6d., post free.

Monthly Parts, 6d.; post free, 7½d.

Ditto, per Ann., 7s. 6d., post free.

### THE HOSPITAL

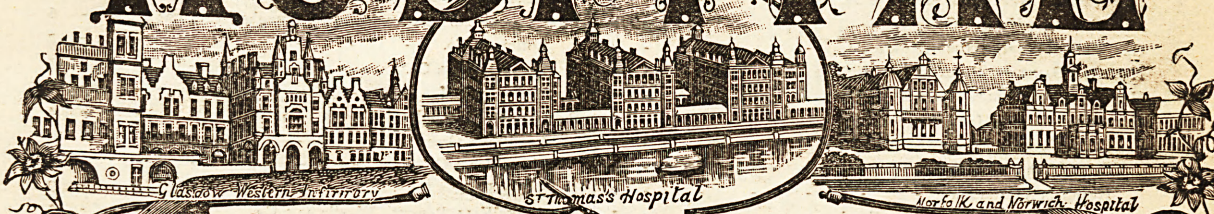

An Institution, Family, and Congregational Journal of

Hospitals, Asylums, & all Agencies for the Care of the Sick, Criticism & News.

